# Internalization of paramagnetic phosphatidylserine-containing liposomes by macrophages

**DOI:** 10.1186/1477-3155-10-37

**Published:** 2012-08-28

**Authors:** Tessa Geelen, Sin Yuin Yeo, Leonie EM Paulis, Lucas WE Starmans, Klaas Nicolay, Gustav J Strijkers

**Affiliations:** 1 Department of Biomedical Engineering, Biomedical NMR, Eindhoven University of Technology, PO Box 513, Eindhoven, MB, 5600, the Netherlands

**Keywords:** Inflammation, Liposomes, MRI, Phosphatidylserine, Cell internalization

## Abstract

**Background:**

Inflammation plays an important role in many pathologies, including cardiovascular diseases, neurological conditions and oncology, and is considered an important predictor for disease progression and outcome. *In vivo* imaging of inflammatory cells will improve diagnosis and provide a read-out for therapy efficacy. Paramagnetic phosphatidylserine (PS)-containing liposomes were developed for magnetic resonance imaging (MRI) and confocal microscopy imaging of macrophages. These nanoparticles also provide a platform to combine imaging with targeted drug delivery.

**Results:**

Incorporation of PS into liposomes did not affect liposomal size and morphology up to 12 mol% of PS. Liposomes containing 6 mol% of PS showed the highest uptake by murine macrophages, while only minor uptake was observed in endothelial cells. Uptake of liposomes containing 6 mol% of PS was dependent on the presence of Ca^2+^ and Mg^2+^. Furthermore, these 6 mol% PS-containing liposomes were mainly internalized into macrophages, whereas liposomes without PS only bound to the macrophage cell membrane.

**Conclusions:**

Paramagnetic liposomes containing 6 mol% of PS for MR imaging of macrophages have been developed. *In vitro* these liposomes showed specific internalization by macrophages. Therefore, these liposomes might be suitable for *in vivo* visualization of macrophage content and for (visualization of) targeted drug delivery to inflammatory cells.

## Background

Inflammation plays a crucial role in many pathologies, including cardiovascular diseases, neurological disorders and oncology, and is generally considered as an important predictor for disease progression and outcome [1,2]. Therefore, modulation of the inflammatory response by dedicated therapy is of particular interest.

The efficacy of traditional therapeutic compounds of low molecular weight is often limited by short blood circulation half-lives and adverse side effects due to non-specific systemic distribution and accumulation. Additionally, it is difficult to obtain quantitative information on the amount of drug accumulating in the diseased tissue. Drug delivery via a nanocarrier system provides an attractive alternative to alleviate these drawbacks. For example, Doxil is a clinically approved nanocarrier system for cancer treatment, which consists of doxorubicin encapsulated in liposomes [3,4]. This formulation limits cardiotoxicity and prolongs the blood circulation half-life compared to free doxorubicin, which results in an enhanced time window for drug delivery and extravasation of the liposomes through the leaky tumor vasculature.

The surface composition of nanocarriers containing drugs can be tailored to tune clearance kinetics, for instance polyethylene glycol (PEG) is often incorporated to prolong the blood half-life [5]. Furthermore, the larger size of nanocarriers promotes a higher level of uptake in diseased tissues by the enhanced permeability and retention (EPR) effect [6,7]. Importantly, to address the inflammatory response in cardiovascular disease, the drug-containing nanocarriers should be delivered with high specificity to inflammatory cells in the diseased tissue. This can be achieved by introducing ligands that mediate nanocarrier recognition and internalization by the inflammatory cells.

An attractive route to target macrophages is by incorporation of the lipid phosphatidylserine (PS) in lipid-based nanoparticles, such as liposomes. In mammalian cells, PS is predominantly present in the inner leaflet of cell membranes. When a cell becomes apoptotic, PS is exposed on the outer leaflet of the cell membrane, which serves as a trigger for phagocytosis by macrophages [8,9]. The incorporation of PS in the liposomal membrane can therefore promote uptake by macrophages. Previously it was shown that incorporation of PS in liposomes indeed resulted in enhanced uptake by macrophages [10,11].

Also, magnetic resonance imaging (MRI) contrast agents can be incorporated to image drug delivery and obtain quantitative information on the local concentration of drugs at the target site [12,13]. Previously, Harel-Ader *et al.* developed liposomes with PS containing iron-oxides for MRI visualization of inflammatory cells in myocardial infarction [14] and Maiseyeu *et al.* described liposomes with PS containing Gd-DTPA-distearylamide for MR imaging of macrophages in atherosclerotic plaques [10]. However, a detailed characterization and optimization of MRI-detectable PS-containing liposomes, including the conditions under which they most effectively target macrophages and induce strongest contrast in MRI, is still lacking.

In this study, we therefore describe the design and characterization of MRI-detectable liposomes that are targeted to macrophages using PS. Liposomes containing different molar percentages of PS were prepared and liposome size and morphology were studied by dynamic light scattering (DLS) and cryogenic transmission electron microscopy (cryoTEM). Fluorescent labels, incorporated in the liposomes, enabled detailed analysis of liposome binding and internalization by macrophages using confocal laser scanning microscopy (CLSM) and fluorescence activated cell sorting (FACS). The ability of the liposomes to induce contrast changes in MR images was studied in macrophages and quantified on the basis of the measured changes in T_1_ and T_2_ relaxation times.

## Results

### Characterization of liposomes

**Figure 1:**
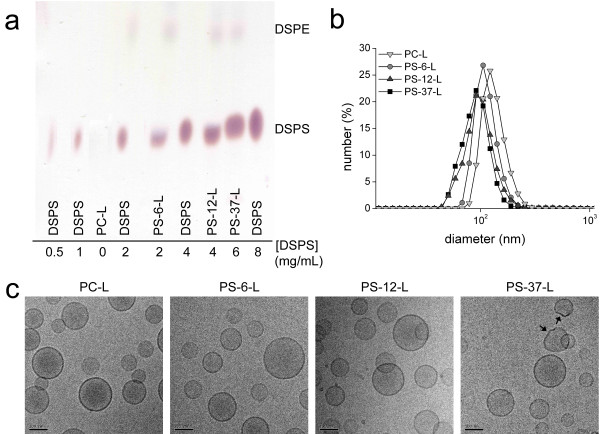
**Characterization of paramagnetic liposomes. a**) TLC of the different types of liposomes. **b**) Representative number-weighted DLS size distributions for all liposome formulations. **c**) cryoTEM images of the liposomes. The arrows point at non-spherical liposomes present in PS-37-L. Scale bar = 100 nm.

Liposomes containing 0, 6, 12 and 37 mol% of phosphatidylserine (1,2-distearoyl-*sn*-glycero-3-phospho-L-serine = DSPS or PS) were prepared. In this paper we will refer to these liposomes as PC-L, PS-6-L, PS-12-L, and PS-37-L, respectively (see also Table 1 and Methods). Thin layer chromatography (TLC) confirmed the presence of PS in the liposome formulations by the appearance of a spot corresponding to DSPS (Figure 1a). The spot became more intense with increasing mol% PS in the lipid preparation mixture, which shows that PS was successfully incorporated in increasing amounts in the final liposome preparations up to 37 mol%.

**Table 1:** Mol% of lipids present in different liposome formulations

	**PC-L**	**PS-6-L**	**PS-12-L**	**PS-37-L**
**DSPS**	0	6	12	37
**DSPC**	37	31	25	0
**Gd-DOTA-DSPE**	25	25	25	25
**Cholesterol**	33	33	33	33
**PEG2000-DSPE**	5	5	5	5

Representative DLS spectra for the different types of liposomes are presented in Figure 1b. For all formulations a single dominant peak was observed, indicating a relatively narrow range of liposome diameters. PS-6-L and PS-12-L had the same mean hydrodynamic diameter as PC-L (Table 2). However, PS-37-L had a somewhat smaller diameter (p <0.05 vs. PC-L). Incorporation of PS resulted in a significant increase of the polydispersity index (PDI, p <0.05 vs. PC-L), which was also observed as a modest broadening of the DLS peaks (Figure 1b). We think that changes in the membrane rigidity or stability due to incorporation of PS leads to a smaller size after extrusion. Liposome morphology was investigated in more detail using cryoTEM (Figure 1c). CryoTEM images revealed predominantly single unilamellar liposomes for all formulations. For PC-L, PS-6-L and PS-12-L liposomes were spherical, whereas for PS-37-L occasionally non-spherical, deformed liposomes were observed (Figure 1c, black arrows).

**Table 2:** Characterization of liposome formulations

	**PC-L**	**PS-6-L**	**PS-12-L**	**PS-37-L**
**Hydrodynamic diameter (nm)**^**a**^	136 ± 3	120 ± 7	111 ± 10	97 ± 4^*^
**PDI (−)**^**a**^	0.13 ± 0.03	0.28 ± 0.05^*^	0.30 ± 0.02^*^	0.30 ± 0.19^*^
**r**_**1**_**(mM**^**-1**^·**s**^**-1**^**)**^**b**^	3.0 ± 0.1	3.0 ± 0.3	3.4 ± 0.4	4.0 ± 0.1
**r**_**2**_**(mM**^**-1**^·**s**^**-1**^**)**^**b**^	42.3 ± 5.5	51.5 ± 4.5	60.7 ± 5.1	46.7 ± 4.2
**r**_**2**_**/r**_**1**_ ^**b**^	14.4 ± 2.0	18.1 ± 2.6	18.2 ± 0.8	11.8 ± 0.9

^a^ n = 5 for PC-L and PS-6-L and n = 4 for PS-12-L and PS-37-L; ^b^ n = 4 for PC-L and PS-6-L, and n = 3 for PS-12-L and PS-37-L; ^*^ p <0.05 vs. PC-L, ANOVA with Bonferroni correction.

The ability of the liposomes to generate contrast in MRI is determined by their potency to change the longitudinal (T_1_) and transversal (T_2_) relaxation times, which is expressed by the longitudinal (r_1_) and transversal (r_2_) relaxivity. The r_1_ and r_2_ of the liposomes at 9.4 T and room temperature, normalized to Gd concentration, were 3.0-4.0 mM^-1^·s^-1^ and 42–60 mM^-1^·s^-1^, respectively (Table 2). Incorporation of PS did not significantly affect the longitudinal and transversal relaxivity. All liposome formulations displayed a similar relatively high r_2_/r_1_ ratio.

### Association of PS-containing liposomes with macrophages

*In vitro* experiments were performed to determine which formulation of PS-containing liposomes resulted in highest association with mouse macrophages (RAW cells). RAW cells were incubated with PC-L, PS-6-L, PS-12-L and PS-37-L and association of liposomes with the macrophages was characterized by several readouts exploiting the various components of the liposomes, including quantitative T_1_ and T_2_ mapping with MRI, quantitative Gd determinations by inductively coupled plasma mass spectrometry (ICP-MS), and CLSM.

**Figure 2:**
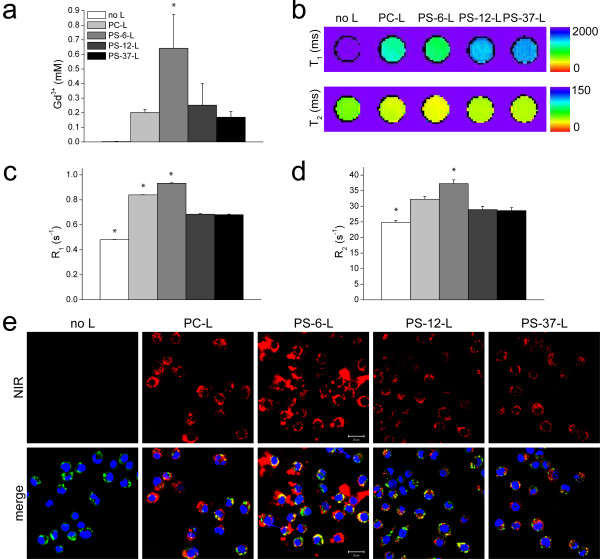
**Association of PS-containing liposomes with RAW cells. a**) Gd concentration of RAW cells incubated with different liposome formulations in RPMI medium as determined with ICP-MS (n = 3/group, except for PS-6-L n = 2/group). * p <0.05 vs. no L, ANOVA with Bonferroni correction. **b**) Representative T_1_ and T_2_ maps of RAW cell pellets. **c**) Average R_1_ and **d**) average R_2_ of cell pellets measured at 9.4 T. * p <0.05 vs. all, ANOVA with Bonferroni correction. **e**) CLSM images of RAW cells. Top row: in red, the fluorescence signal of the NIR-lipids present in liposomes (laser intensity 2% of maximal intensity). Bottom row: NIR signal (in red) merged with signal of labeled macrophage CD68 (in green) and cell nuclei (in blue). Scale bar = 20 μm.

Figure 2a shows results of quantitative Gd determinations of RAW cells, including untreated cells (no L) and cells incubated with PC-L, PS-6-L, PS-12-L or PS-37-L. Gd concentrations for the incubations with PS-12-L and PS-37-L were at the baseline level of PC-L non-specific uptake and untreated cells. Solely, incubation with PS-6-L resulted in a significantly higher Gd concentration (0.64 ± 0.23 mM, p <0.05 vs. no L).

MRI measurements were performed at 9.4 T on the same cell pellets as for ICP-MS. MRI consisted of quantitative T_1_ and T_2_ mapping. The cells incubated with PS-6-L could clearly be distinguished from untreated cells and cells incubated with other types of liposomes (Figure 2b). Average R_1_ (=1/T_1_) and R_2_ (=1/T_2_) values for the different groups are summarized in Figure 2c and 2d, respectively. Incubation with liposomes always resulted in enhanced R_1_ and R_2_ values (p <0.05 vs. no L). In agreement with quantitative Gd determinations, however less pronounced, both R_1_ and R_2_ were highest for the incubations with PS-6-L (0.942 ± 0.004 s^-1^ and 37.3 ± 1.2 s^-1^, respectively, p <0.05 vs. all).

The relaxivities r_1_ and r_2_ in the cellular environment were estimated from the quantitative Gd determinations in relation to changes in R_1_ and R_2_ (Table 3). For PC-L, r_1_ and r_2_ were 1.9 ± 0.3 mM^-1^·s^-1^ and 32.5 ± 3.6 mM^-1^·s^-1^, respectively. For PS-6-L, r_1_ and r_2_ were 0.8 ± 0.4 mM^-1^·s^-1^ and 16.3 ± 5.8 mM^-1^·s^-1^. As shown previously, PC-L and PS-6-L relaxivities in aqueous solution were similar (Table 2). The lower cellular relaxivities for PS-6-L compared to PC-L therefore suggested a different PS-6-L uptake mechanism in RAW cells and consequently a different cellular distribution, which was investigated in more detail as described further on.

**Table 3:** Relaxivities of the liposomes in the cellular environment

	**Cellular r**_**1 **_**(mM**^**-1 **^**·s**^**-1**^**)**	**Cellular r**_**2 **_**(mM**^**-1 **^**·s**^**-1**^**)**
**PC-L**	1.9 ± 0.3	32.5 ± 3.6
**PS-6-L**	0.8 ± 0.4	16.3 ± 5.8
**PS-12-L**	1.4 ± 0.5	19.1 ± 6.9
**PS-37-L**	1.3 ± 0.1	22.0 ± 8.1

CLSM imaging of the near-infrared (NIR)-labeled lipids incorporated in the liposomal membrane revealed association of all types of liposomes with RAW cells (Figure 2e). No NIR autofluorescence signal was detected in RAW cells incubated without liposomes. In agreement with MRI, NIR fluorescence and therefore liposome association was highest for cells incubated with PS-6-L and intermediate for PC-L, while PS-12-L and PS-37-L showed similarly low levels of NIR fluorescence.

**Figure 3:**
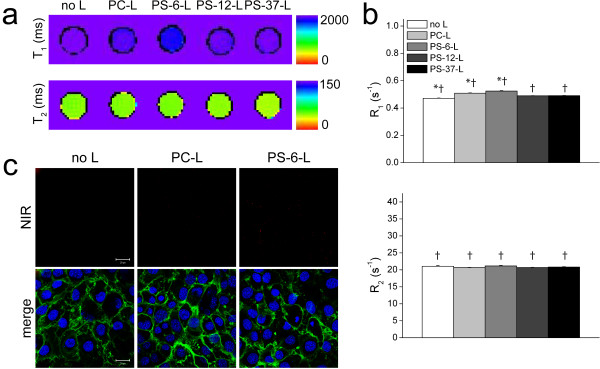
**Association of PS-containing liposomes with H5V cells. a**) Representative T_1_ and T_2_ maps of H5V cell pellets incubated with the different types of liposomes in RPMI medium. **b**) Average R_1_ and R_2_ of H5V cell pellets measured at 9.4 T (n = 3/group). * p <0.05 vs. all, ANOVA with Bonferroni correction and † p <0.05 vs. RAW cell pellets, Student’s t-test. **c**) CLSM images of H5V cells. Top row: in red, the fluorescence signal of the NIR-lipids present in liposomes. The laser intensity (20% of maximal intensity) was ten times higher as compared to Figure 2d. Bottom row: NIR signal (in red) merged with signal of labeled H5V cell membranes (in green) and cell nuclei (in blue). Scale bar = 20 μm.

To confirm that the observed association of PS-6-L with RAW cells was mediated by their phagocytic character, endothelial H5V cells, for which no or minor phagocytosis was expected, were incubated with the different liposome formulations. MRI T_1_ and T_2_ maps for untreated and liposome-incubated cell pellets, shown in Figure 3a, revealed only minor differences between the various groups. Nevertheless, incubation with liposomes resulted in enhanced R_1_ values (p <0.05 vs. no L) and the highest values were detected for cells incubated with PS-6-L (0.524 ± 0.003 s^-1^, p <0.05 vs. all, Figure 3b). No significant differences were detected for R_2_ (p >0.05 vs. all). However, R_1_ and R_2_ values were significantly lower compared to RAW cell incubations for all groups (p <0.05 vs. RAW cells). ΔR_1_ (= R_1,PS-6-L_ - R_1,no L_) values were 8.5 times lower for H5V cells compared to RAW cells, and for ΔR_2_ this was even 45 times. CLSM of the H5V cells revealed no NIR fluorescence for incubations with PC-L and very few faint spots for PS-6-L (Figure 3c).

### Divalent cation dependency of liposome association with macrophages

The above-described experiments suggested that liposomes with 6 mol% DSPS (PS-6-L) were the most optimal formulation for targeting of macrophages. Therefore, PS-6-L was used in the experiments described from here. Association of PS-containing vesicles with the macrophage cell membrane depends on the presence of divalent cations such as Ca^2+^ and Mg^2+^[11]. To test whether PS-6-L binding depended on the presence of divalent cations, which could be indicative for an interaction between these ions and PS resulting in membrane binding, RAW cells were incubated with PC-L and PS-6-L in Hank’s buffered salt solution (HBSS) with 1.26 mM Ca^2+^ and 0.90 mM Mg^2+^ (HBSS+) or without Ca^2+^ and Mg^2+^ (HBSS-). Furthermore, samples from RAW cells incubated in incubation medium with intermediate Ca^2+^ and Mg^2+^ concentrations (0.424 mM and 0.407 mM, respectively), identical to the ones used in previous experiments, were included. Cellular association was quantified with FACS.

**Figure 4:**
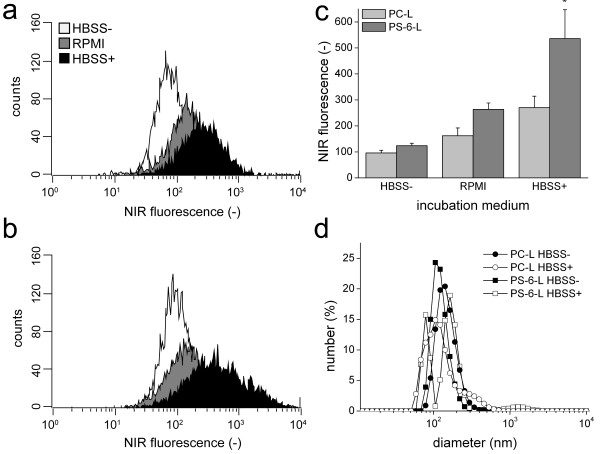
**Divalent cation dependency of liposome association with RAW cells. **Representative FACS spectra for RAW cells incubated with **a**) PC-L or **b**) PS-6-L in HBSS- without Mg^2+^ and Ca^2+^, RPMI incubation medium (0.424 mM Ca^2+^ and 0.407 mM Mg^2+^) or HBSS+ with 1.26 mM Mg^2+^ and 0.90 mM Ca^2+^. **c**) Average fluorescence intensities for all samples (n = 4/group). * p <0.05 vs. all, ANOVA with Bonferroni correction. **d**) Number-weighted DLS size distributions for PC-L and PS-6-L incubated in medium with and without Ca^2+^ and Mg^2+^ (2 h, 37°C).

With increasing Ca^2+^ and Mg^2+^ concentrations, the FACS fluorescence intensities of RAW cells increased for both PC-L and PS-6-L (Figure 4a and 4b, respectively). The average NIR fluorescence for cells incubated with PC-L or PS-6-L in medium lacking Ca^2+^ and Mg^2+^ (HBSS-) was equal (p >0.05, Figure 4c). Importantly, for incubations in medium with high Ca^2+^ and Mg^2+^ concentrations (HBSS+), fluorescence was significantly highest for PS-6-L (p <0.05 vs. all).

DLS showed that the diameter of both PC-L and PS-6-L increased after 2 h of incubation in HBSS+(Figure 4d). For PC-L the average hydrodynamic diameter changed from 164.3 ± 0.9 nm in HBSS- to 209.5 ± 25.2 nm, while for PS-6-L the diameter increased from 104.6 ± 19.4 nm to 170.8 ± 33.5 nm. This size increase could additionally enhance the uptake of both types of liposomes by the cells.

### Binding versus internalization

**Figure 5:**
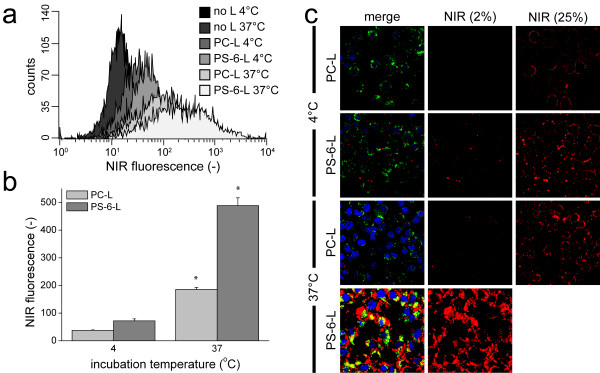
**Internalization versus binding. a**) Representative FACS spectra for RAW cells incubated in HBSS+ at 4°C or 37°C with PC-L or PS-6-L, or without liposomes. **b**) Average fluorescence intensities for all samples (n = 3/group). * p <0.05 vs. all, ANOVA with Bonferroni correction. **c**) CLSM of RAW cells. In red the fluorescence of NIR-lipids present in liposomes, in green macrophage CD68 and in blue cell nuclei are shown. For the NIR signal two different laser intensities are shown (2% and 25% of maximal laser intensity). Scale bar = 20 μm.

To study whether PS-6-L were internalized by macrophages, RAW cells were incubated with PC-L or PS-6-L in HBSS+ at either 4°C or 37°C. Incubation at 4°C inhibits phagocytosis and thus a comparison between 4°C or 37°C enabled a differentiation between binding to the cell membrane and internalization. FACS analysis of cells incubated at 4°C revealed no significant differences in average fluorescence intensities after incubation with PC-L and PS-6-L (Figure 5a-b). At 37°C, however, a significantly higher fluorescence intensity was observed for PS-6-L (p <0.05 vs. all, Figure 5a-b).

CLSM confirmed the FACS measurements (Figure 5c). Incubation of RAW cells with PC-L and PS-6-L at 4°C resulted in minor association of liposomes. CLSM using higher laser intensities showed that the liposomes appeared as a rim around every cell, bound to the cell membrane. No significant internalization was observed. For incubations with PC-L at 37°C CLSM images were comparable to incubations at 4°C, with minor association of liposomes, and higher laser intensities revealed that PC-L were mainly bound to the cell membrane. CLSM confirmed that incubation with PS-6-L at 37°C resulted in massive internalization of the liposomes, as shown by the high NIR signal inside RAW cells.

## Discussion

Macrophages play a decisive role in several cardiovascular diseases. For example, in atherosclerosis high macrophage content is one of the hallmarks of plaque vulnerability [2]. The inflammatory response after myocardial infarction is important for cardiac remodeling and outcome [1]. Therefore, macrophages form a significant therapeutic target in cardiovascular diseases and tools for noninvasive MR imaging of macrophages are highly desired. Iron oxides have been successfully applied for the MR visualization of macrophages in cardiovascular diseases [15-17]. Nevertheless, targeting of iron oxides to CD11b/CD18, which is expressed on macrophages, did not improve specificity for MR imaging of macrophages in a mouse model of atherosclerosis [18]. Recently, Gd-labeled liposomes were used to visualize monocytes and/or macrophages infiltration in the mouse myocardium up to 7 days after myocardial infarction [19].

In this study, we describe the design and characterization of paramagnetic liposomes targeted to macrophages by incorporation of PS in the liposomal membrane. The liposomes contained Gd-DOTA-DSPE for MRI detection. Gd-DOTA-DSPE is a phospholipid that presents a high r_1_ and the Gd-DOTA complex displays a high thermodynamic and kinetic stability [20]. As expected, at 9.4 T, the longitudinal relaxivity is not as high as at lower, clinical field strengths [20,21]. Importantly, incorporation of PS did not significantly affect liposomal r_1_ and r_2_ values. The r_2_/r_1_ ratio of the liposome formulations at 9.4 T was relatively high, which means that the liposomes will display a significant T_2_ effect as well. Nevertheless, by appropriately choosing the MRI sequence parameters, the T_1_ effect of the liposomes can be effectively exploited (Figure 2).

A distinct difference between the PS-containing liposomes used in this study and previously reported formulations for use in *in vivo* MRI studies is the incorporation of 5 mol% polyethylene glycol (PEG) lipids in the liposomal membrane. PEG reduces the interactions between the liposomes, reducing aggregation and ensuring a monodisperse formulation (Table 2 and Figure 1). Additionally, PEG increases the *in vivo* blood circulation half-life by reducing the interactions with plasma proteins, assuring a longer interaction time with macrophages [5]. According to previous studies, incorporation of 5 mol% PEG in PS-containing liposomes is not impeding the interaction of PS with macrophages, since at least 10–15 mol% PEG would be needed to completely shield the liposomes from any interactions with proteins [22-24]. We therefore did not expect a decrease in the uptake by shielding of the PS.

Liposomes containing 6 mol% PS resulted in the highest uptake by RAW murine macrophages (Figure 2). Maiseyeu *et al.* and Rimle *et al.* have observed optimal uptake by macrophages of liposomes without PEG when these contained 5–12 mol% PS [10,11]. Interestingly, these experimentally determined optimal concentrations are in the range of 2–10 mol% PS found in the membranes of mammalian cells [25], which suggests that macrophages are optimally equipped to recognize and phagocytose nanoparticles that express approximate physiological concentrations of PS. Association was specific for macrophages as uptake by endothelial H5V cells was significantly lower (Figure 3).

Uptake of PS-containing liposomes by macrophages was stimulated by the presence of divalent cations (Figure 4). Higher uptake was not primarily caused by divalent cation-mediated clustering of the liposomes, since incubation of liposomes in HBSS+ resulted in moderate changes in liposome size for both PC-L and PS-6-L. The HBSS+ buffer contained a physiologically relevant concentration of 1.26 mM Ca^2+^, compared to for example approximately 1.24 mM Ca^2+^ in mouse blood [26]. For the PS-mediated recognition of apoptotic cells by macrophages, different engulfment receptors have been identified, such as scavenger receptors, oxidized low-density lipoproteins recognizing receptors and CD68 [27], which for the LOX-1 scavenger receptor has been proven to be Ca^2+^-dependent [28]. Which of these receptors are important for PS-mediated uptake of liposomes remains unknown.

With respect to MR imaging of liposome uptake, a relatively high association of PS-6-L with macrophages, as determined with ICP-MS, resulted only in a modest increase in R_1_ (Figure 2). This is probably related to compartmentalization of PS-6-L in intracellular vesicles after phagocytosis, which limits effective access of bulk water protons to the Gd contrast agent [29,30]. T_1_ shortening requires direct physical contact between Gd and water protons to be most effective. This interpretation is corroborated by the observation that the estimated cellular relaxivity of PS-6-L (r_1_ = 0.8 ± 0.4 mM^-1^·s^-1^) was lower than the one of PS-6-L in aqueous solution (r_1_ = 3.0 ± 0.3 mM^-1^·s^-1^). Furthermore, internalization of PS-6-L was observed by CLSM for incubations at 37°C (Figure 5).

The next step will be to apply and study the uptake of PS-6-L in a relevant animal model of cardiovascular inflammation, for example in atherosclerosis or myocardial infarction. Christiansen *et al.* have shown that echocardiography of PS-containing microbubbles trapped in infarcted myocardium correlated moderately well with MPO activity, which are excreted by inflammatory cells [31].

Apart from use in imaging applications, PS-containing liposomes are a promising vehicle for targeted drug delivery. Liposomes loaded with Q10, ATP or adenosine delivered to infarct myocardium were demonstrated to reduce infarct size and salvage ischemic myocardium [32-34]. Also, liposomes have been used as a vehicle for delivery of glucocorticoids drugs to perform anti-inflammatory cancer therapy [12]. Targeting could enhance the specificity of drug delivery to macrophages. Alternatively, PS-liposomes themselves can be used for therapy of inflammation as well [14,35-37]. As PS-liposomes mimic apoptotic cells, they inhibit pro-inflammatory cytokines release and promote secretion of anti-inflammatory cytokines. However, for therapy purposes higher PS-concentrations (up to 30 mol%) were used [14,36], which in this study did not enhance uptake by macrophages.

## Conclusions

In summary, paramagnetic liposomes, containing 6 mol% of PS, showed enhanced uptake by macrophages compared to liposomes without PS, while significantly less uptake was observed for non-phagocytic cells. Association of PS-containing liposomes to macrophages was increased by the presence of divalent cations in the incubation medium and resulted mainly in internalization of liposomes, whereas only minor binding was observed. Therefore, these liposomes can be used for molecular MR imaging of macrophages and might as well be suitable for targeted drug delivery to macrophages in cardiovascular diseases.

## Methods

### Preparation of PS-containing liposomes

Liposomes containing different mole percentages of PS were prepared by modification of the protocol described by Hak *et al.*[20]. In short, lipid film hydration of a lipid mixture was performed (typically 50 μmol of total lipid). The lipid mixture, consisting of 1,2-distearoyl-*sn*-glycero-3-phospho-L-serine (DSPS, Avanti Polar Lipids, Alabaster, USA), 2-distearoyl-*sn*-glycero-3-phosphocholine (DSPC, Lipoid, Steinhausen, Switzerland), Gd-DOTA-1,2-distearoyl-*sn*-glycero-3-phospoethanolamine (Gd-DOTA-DSPE, SyMO-Chem BV, Eindhoven, the Netherlands), cholesterol (Avanti Polar Lipids) and 1,2-distearoyl-*sn*-glycero-3-phospoethanolamine-*N*-[methoxy(poly(ethylene glycol))-2000] (PEG2000-DSPE, Lipoid), was dissolved in chloroform and methanol (8:1 v/v) at molar percentages as shown in Table 1. For liposomes containing DSPS, the mixture was heated to dissolve the DSPS (to maximally 65°C). Additionally, 0.1 mol% near-infrared664-1,2-distearoyl-*sn*-glycero-3-phospoethanolamine (NIR664-DSPE, SyMO-Chem BV) was incorporated. After rotary evaporation at 30°C and overnight drying under a nitrogen flow, the lipid film was hydrated in HEPES-buffered saline (HBS, 10 mM HEPES, 135 mM NaCl, pH 7.4) at 65°C. The resulting multilamellar vesicles were sized by extrusion through 400 nm filters (2 times) and 200 nm filters (8 times). Finally, the liposomes were concentrated using ultracentrifugation (45 min, 55,000 rpm, 4°C) and resuspended in HBS at a concentration of approximately 70 mM total lipid.

### Characterization of liposomes

Total lipid concentrations of the final liposome formulations were determined by a phosphate determination according to Rouser [38]. Hydrodynamic number-weighted size and size distribution were assessed with dynamic light scattering (DLS, ZetaSizer NanoS, Malvern Instruments, Worcestershire, UK) at 23°C.

To confirm the presence of DSPS lipids in the PS-containing liposomes, thin layer chromatography (TLC) was performed on an aluminum sheet coated with silica gel 60 F_254_ (Merck BV, Schiphol-Rijk, the Netherlands) [39]. As eluent a mixture of chloroform, methanol, glacial acetic acid and water (65:25:8:4 v/v) was used. Liposomes were applied (expected concentrations of DSPS: PC-L 0 mg/mL, PS-6-L 2 mg/mL, PS-12-L 4 mg/mL and PS-37-L 6 mg/mL) and allowed to migrate for 30 min. As controls standard solutions of DSPS (0.5, 1, 2, 4 and 8 mg/mL) were used. Finally, primary and secondary amines in DSPS and Gd-DOTA-DSPE were detected with ninhydrin.

Liposomal morphology was evaluated with cryogenic transmission electron microscopy (cryoTEM). Samples were vitrified on carbon-coated cryoTEM grids with a vitrification robot (Vitrobot Mark III, FEI, Hillsboro, USA). Imaging was performed on a Tecnai 20 Sphera TEM instrument (FEI) equipped with a LaB6 filament (200 kV) and Gatan cryoholder (approximately -170°C) at 25,000x magnification.

Liposomal longitudinal and transversal relaxation times (T_1_ and T_2_) were determined with a 9.4 T small animal MR scanner (Bruker Biospin GmbH, Ettlingen, Germany) equipped with a 35-mm-diameter quadrature birdcage RF coil (Rapid Biomedical, Rimpar, Germany). For T_1_ measurements an inversion recovery fast low angle shot (FLASH) sequence was used, with the following parameters: overall repetition time (TR) 15 sec, TR 4 ms, echo time (TE) 2 ms, flip angle (α) 15^o^, number of excitations (NEX) 4, field of view (FOV) 3x3 cm^2^, matrix 128x128, 1 mm slice thickness, 32 segments and 60 inversion times ranging from 72 to 4792 ms. T_2_ relaxation times were determined using a multi-slice multi-echo sequence with the following parameters: TR 2000 ms, 32 TEs ranging from 9 to 288 ms, α 180^o^, NEX 4, FOV 3x3 cm^2^, matrix 128x128 and 1 mm slice thickness. T_1_ and T_2_ relaxation times were calculated by mono-exponential fitting with a custom-built fitting program (Mathematica 6, Wolfram Research Europe, Oxfordshire, UK). Relaxivities r_1_ and r_2_ (in mM^-1^·s^-1^) were determined from R_i_ = R_i,0_ + r_i_·[Gd], with i = 1,2, R_i_ = 1/T_i_, R_i,0_ the relaxation rate of a sample without liposomes and [Gd] between 0.001 and 1 mM Gd.

### Cell culture

Mouse macrophages, RAW264.7 (European Collection of Animal Cell Cultures (ECACC)), were cultured in RPMI medium (phenol-red free), supplemented with 10% FBS, 2 mM L-glutamine and 100 U/mL penicillin/streptomycin. As non-phagocytic control cells, murine heart endothelioma cells, H5V (kindly provided by prof. dr. G. Molema, University of Groningen, the Netherlands), were maintained in DMEM medium, containing 10% FBS, 2 mM L-glutamine and 100 U/mL penicillin/streptomycin [40].

### Association of PS-containing liposomes with RAW cells

To determine the mol% of PS present in liposomes resulting in maximal uptake by macrophages, RAW cells were incubated with PC-L, PS-6-L, PS-12-L and PS-37-L for 2 h at 37°C (1 mM total lipid). For MRI and inductively coupled plasma mass spectrometry (ICP-MS), cells were harvested by scraping and non-bound liposomes were removed by centrifugation (3x5 min, 500 g, RPMI medium at 37°C). Cells were fixed in 4% PFA (250 μL) and a loosely packed cell pellet was allowed to form by storage at 4°C (>2 days). For confocal laser scanning microscopy (CLSM), cells were cultured on coverslips. After incubation with the liposome formulations, cells were fixed with 4% PFA (20 min). Finally cells were washed with and stored in phosphate bufferd saline (PBS).

### Association of PS-containing liposomes with H5V cells

To confirm that PS-containing liposomes were not taken up by endothelial cells, H5V cells were incubated with PC-L, PS-6-L, PS-12-L and PS-37-L for 2 h at 37°C (1 mM total lipid). For MRI, cells were washed with medium (37°C) and PBS (37°C). Afterwards, cells were harvested with trypsin/EDTA, fixed with 4% PFA and a loosely packed pellet was allowed to form. For CLSM, cells were cultured on gelatin-coated coverslips and handled as described above.

### Divalent cation dependency of liposome association with RAW cells

The association of liposomes to RAW cells under different calcium and magnesium concentrations was studied. RAW cells were incubated with PC-L and PS-6-L (2 h, 37°C, 1 mM total lipid) in Hank’s buffered salt solution (HBBS) containing 1.26 mM Ca^2+^ and 0.90 mM Mg^2+^ (HBSS+), HBSS without Ca^2+^ and Mg^2+^ (HBSS-) and RPMI medium (0.424 mM Ca^2+^ and 0.407 mM Mg^2+^). Afterwards, cells were harvested by scraping, washed in the appropriate medium (HBSS+, HBSS- or RPMI, 37°C), fixed in 4% PFA (20 min) and stored in 0.01% sodium-azide in PBS for FACS.

To investigate possible clustering of PC-L and PS-6-L under high calcium and magnesium concentrations, liposomes were incubated in HBSS+ or HBSS- (2 h, 37°C). Changes in hydrodynamic number-weighted diameter and size distribution were measured with DLS as described above at 37°C.

### Binding versus internalization

To evaluate phagocytosis of PS-containing liposomes by macrophages, RAW cells were incubated with PC-L or PS-6-L at 4°C or at 37°C (1 mM total lipid in HBSS+, 2 h). Incubation at 4°C inhibits phagocytosis. For FACS, cells were incubated with liposomes in HBSS+ and harvested and washed as described above. For CLSM, cells were cultured and incubated with liposomes in microscopy chambers (Ibidi GmbH, München, Germany). Afterwards, cells were washed with HBSS+ (4°C or 37°C), fixed with 4% PFA (20 min), washed and stored in PBS.

### Cellular relaxation rates and relaxivities

The cellular relaxation rates of cell pellets (R_1_ and R_2_) were determined at 9.4 T using the MRI protocol as described above. Furthermore, the cell pellet volume was determined using a 3D FLASH sequence with the following parameters: TR 25 ms, TE 3.7 ms, α 30^o^, NEX 1, FOV 25.6x25.6x25.6 mm^3^ and matrix 256x256x256. Cell pellets were segmented with OsiriX Imaging Software (http://www.osirix-viewer.com) and pellet volumes were calculated. The Gd content of cell pellets was determined with ICP-MS (DRCII, Perkin Elmer, Waltham, USA) after destruction in nitric acid and perchloric acid (1:2 v/v) at 180°C. Next, gadolinium concentrations were derived using the cell pellet volume. Cellular relaxivities were calculated from R_i_ = R_i,0_ + r_i_·[Gd], with i = 1,2, and R_i,0_ the relaxation rate of untreated cells.

### Cellular fluorescence quantification

The fluorescence intensity of NIR664-lipids, present in the liposomes associated with RAW cells, was quantified by fluorescence activated cell sorting (FACS) on a Guava Easycyte 8HT (Millipore, Billerica, USA). NIR664 was excited with a 640 nm laser and detected using a 661/19 nm band-pass filter. Mean cellular fluorescence intensity was calculated with GuavaSoft 1.0 software (Millipore) and was corrected for autofluorescence as detected by measurement of untreated cells.

### Cellular CLSM

The cellular distribution of liposomes was studied with CLSM. Cell membranes of RAW cells were labeled with rat anti-mouse CD68-fluorescein isothiocynate (CD68-FITC, 4 μg/mL, AbD Serotec, Dusseldorf, Germany). H5V cells were labeled with rat anti-mouse CD31 (10 μg/mL, BioLegend, Uithoorn, The Netherlands) conjugated to goat anti-rat FITC (Invitrogen, Bleiswijk, The Netherlands). Nuclei were stained with 4′6-diamidino-2-phenylindole dihydrochloride (DAPI, 0.1 μg/mL, Invitrogen).

A Zeiss LSM META system (Carl Zeiss BV, Sliedrecht, the Netherlands) was used for acquisition of CLSM images. NIR664 present in liposomes was excited with a 633 nm HeNe laser (5.0 mW) and the emission was filtered with a 680/60 nm band-pass filter. Cell membranes labeled with FITC were excited with a 488 nm Ar laser and the emission was filtered with a 525/50 nm band-pass filter. For two-photon excitation of DAPI, a Ti:Sapphire laser tuned to 780 nm was used and emission was captured with a 460/50 band-pass filter. All images were acquired with a 63x/1.4 oil immersion objective, a matrix of 2048x2048, resulting in a resolution of 0.07x0.07 μm^2^, and 4 averages.

### Statistics

All data are presented as mean ± standard error of the mean (SEM). To test for significant differences between groups, one-way analysis of variance (ANOVA) with Bonferroni correction for multiple group comparisons or a Student’s t-test for independent samples was applied. All statistical analyses were performed in PASW Statistics 18.02 (IBM Corporation, Armonk, NY, USA) and P <0.05 was considered significant.

## Abbreviations

ANOVA: Analysis of variance; CLSM: Confocal laser scanning microscopy; cryoTEM: Cryogenic transmission electron microscopy; DAPI: 4′6-diamino-2-phenylindole dihydrochloride; DSPC: 1,2-distearoyl-*sn*-glycero-3-phospocholine; DSPS: 1,2-distearoyl-*sn*-glycero-3-phospho-L-serine; DSPE: 1,2-distearoyl-*sn*-glycero-3-phospoethanolamine; DLS: Dynamic light scattering; EPR: Enhanced permeability and retention; FACS: Fluorescence activated cell sorting; FBS: Fetal bovine serum; FITC: Fluorescein isothiocynate; Gd: Gadolinium; HBS: HEPES buffered saline; HBSS: Hank’s buffered salt solution; HBSS-: HBSS without Ca^2+^ and Mg^2+^; HBSS+: HBSS with Ca^2+^ and Mg^2+^; ICP-MS: Inductively coupled plasma mass spectrometry; L: Liposomes; MRI: Magnetic resonance imaging; NIR: Near-infrared; PBS: Phosphate buffered saline; PC: Phosphatidylcholine; PDI: Polydispersity index; PEG: Polyethylene glycol; PFA: Paraformaldehyde; PS: Phosphatidylserine; PS-x-L: Liposomes containing x mol% of PS; r_1_: Longitudinal relaxivity; r_2_: Transversal relaxivity; R_1_: Longitudinal relaxation rate; R_2_: Transversal relaxation rate; SEM: Standard error of the mean; T_1_: Longitudinal relaxation time; T_2_: Transversal relaxation time; TLC: Thin layer chromatography.

## Competing interests

The authors declare that they have no competing interests.

## Authors’ contributions

All authors added intellectual content, read and approved the final manuscript. TG: Designed the study, performed experiments, performed data analysis, performed statistical analysis, prepared and edited the manuscript. SY, LP, LS: Performed experiments, performed data analysis, edited the manuscript. KN: Co-designed the study, edited the manuscript. GS: Principal investigator, designed the study, prepared and edited the manuscript.

## References

[B1] Frangogiannis NG The immune system and cardiac repair Pharmacol Res 2008 58 88 111 10.1016/j.phrs.2008.06.007 18620057 PMC2642482

[B2] Glass CK Witztum JL Atherosclerosis: the road ahead Cell 2001 104 503 516 10.1016/S0092-8674(01)00238-0 11239408

[B3] Abraham SA Waterhouse DN Mayer LD Cullis PR Madden TD Bally MB The liposomal formulation of doxorubicin Methods Enzymol 2005 391 71 97 15721375 10.1016/S0076-6879(05)91004-5

[B4] Gabizon A Shmeeda H Barenholz Y Pharmacokinetics of pegylated liposomal doxorubicin: review of animal and human studies Clin Pharmacokinet 2003 42 419 436 10.2165/00003088-200342050-00002 12739982

[B5] Allen TM Long-circulating (sterically stabilized) liposomes for targeted drug delivery Trends Pharmacol Sci 1994 15 215 220 10.1016/0165-6147(94)90314-X 7940982

[B6] van Bochove GS Paulis LEM Segers D Mulder WJM Krams R Nicolay K Strijkers GJ Contrast enhancement by differently sized paramagnetic MRI contrast agents in mice with two phenotypes of atherosclerotic plaque Contrast Media Mol Imaging 2011 6 35 45 10.1002/cmmi.402 20882509

[B7] Geelen T Paulis LE Coolen BF Nicolay K Strijkers GJ Passive targeting of paramagnetic lipid-based contrast agents to acute mouse cardiac ischemia/reperfusion injury Proceedings of the 19th Annual Meeting ISMRM; Montreal, Canada 2011 1356

[B8] Hengartner MO The biochemistry of apoptosis Nature 2000 407 770 776 10.1038/35037710 11048727

[B9] Fadok VA Voelker DR Campbell PA Cohen JJ Bratton DL Henson PM Exposure of phosphatidylserine on the surface of apoptotic lymphocytes triggers specific recognition and removal by macrophages J Immunol 1992 148 2207 2216 1545126

[B10] Maiseyeu A Mihai G Kampfrath T Simonetti OP Sen CK Roy S Rajagopalan S Parthasarathy S Gadolinium containing phosphatidylserine liposomes for molecular imaging of atherosclerosis J Lipid Res 2009 50 2157 2163 10.1194/jlr.M800405-JLR200 19017616 PMC2759821

[B11] Rimle D Derski W Petty HR Enhanced binding of phosphatidylserine-containing lipid vesicle targets to RAW264 macrophages Mol Cell Biochem 1984 64 81 87 10.1007/BF00420931 6493224

[B12] Strijkers GJ Kluza E van Tilborg GAF van der Schaft DWJ Griffioen AW Mulder WJM Nicolay K Paramagnetic and fluorescent liposomes for target-specific imaging and therapy of tumor angiogenesis Angiogenesis 2010 13 161 173 10.1007/s10456-010-9165-1 20390447 PMC2911540

[B13] Kluza E Yeo SY Schmid S van der Schaft DWJ Boekhoven RW Schiffelers RM Storm G Strijkers GJ Nicolay K Anti-tumor activity of liposomal glucocorticoids: the relevance of liposome-mediated drug delivery, intratumoral localization and systemic activity J Control Release 2011 151 10 17 10.1016/j.jconrel.2010.11.031 21130819

[B14] Harel-Adar T Ben Mordechai T Amsalem Y Feinberg MS Leor J Cohen S Modulation of cardiac macrophages by phosphatidylserine-presenting liposomes improves infarct repair Proc Natl Acad Sci U S A 2011 108 1827 1832 10.1073/pnas.1015623108 21245355 PMC3033268

[B15] Yang Y Yang Y Yanasak N Schumacher A Hu TC-C Temporal and noninvasive monitoring of inflammatory-cell infiltration to myocardial infarction sites using micrometer-sized iron oxide particles Magn Reson Med 2010 63 33 40 19953508 10.1002/mrm.22175

[B16] Sosnovik DE Nahrendorf M Deliolanis N Novikov M Aikawa E Josephson L Rosenzweig A Weissleder R Ntziachristos V Fluorescence tomography and magnetic resonance imaging of myocardial macrophage infiltration in infarcted myocardium in vivo Circulation 2007 115 1384 1391 10.1161/CIRCULATIONAHA.106.663351 17339546

[B17] Jaffer FA Libby P Weissleder R Molecular Imaging of cardiovascular disease Circulation 2007 116 1052 1061 10.1161/CIRCULATIONAHA.106.647164 17724271

[B18] von zur Muhlen C Fink-Petri A Salaklang J Paul D Neudorfer I Berti V Merkle A Peter K Bode C von Elverfeldt D Imaging monocytes with iron oxide nanoparticles targeted towards the monocyte integrin MAC-1 (CD11b/CD18) does not result in improved atherosclerotic plaque detection by *in vivo* MRI Contrast Media Mol Imaging 2010 5 268 275 10.1002/cmmi.384 20973112

[B19] Naresh NK Xu Y Klibanov AL Vandsburger MH Meyer CH Leor J Kramer CM French BA Epstein FH Monocyte and/or macrophage infiltration of heart after myocardial infarction: MR imaging by using T_1_-shortening liposomes Radiology 2012 264 428 435 10.1148/radiol.12111863 22723500 PMC3401349

[B20] Hak S Sanders HMHF Agrawal P Langereis S Grüll H Keizer HM Arena F Terreno E Strijkers GJ Nicolay K A high relaxivity Gd(III)DOTA-DSPE-based liposomal contrast agent for magnetic resonance imaging Eur J Pharm Biopharm 2009 72 397 404 10.1016/j.ejpb.2008.09.017 18940253

[B21] Strijkers GJ Mulder WJM Van Heeswijk RB Frederik PM Bomans P Magusin PCMM Nicolay K Relaxivity of liposomal paramagnetic MRI contrast agents MAGMA 2005 18 186 192 10.1007/s10334-005-0111-y 16155762

[B22] Chiu G Bally M Mayer L Selective protein interactions with phosphatidylserine containing liposomes alter the steric stabilization properties of poly(ethylene glycol) Biochim Biophys Acta 2001 1510 56 59 10.1016/S0005-2736(00)00335-7 11342147

[B23] Johnstone SA Masin D Mayer L Bally MB Surface-associated serum proteins inhibit the uptake of phosphatidylserine and poly(ethylene glycol) liposomes by mouse macrophages Biochim Biophys Acta 2001 1513 25 37 10.1016/S0005-2736(01)00292-9 11427191

[B24] Levchenko TS Rammohan R Lukyanov AN Whiteman KR Torchilin VP Liposome clearance in mice: the effect of a seperate and combined presence of surface charfe and polymer coating Int J Pharmaceut 2002 240 95 102 10.1016/S0378-5173(02)00129-1 12062505

[B25] Vance JE Steenbergen R Metabolism and functions of phosphatidylserine Prog Lipid Res 2005 44 207 234 10.1016/j.plipres.2005.05.001 15979148

[B26] Tordoff M Bachmanov A Reed D Forty mouse strain survey of voluntary calcium intake, blood calcium, and bone mineral content Physiol Behav 2007 91 632 643 10.1016/j.physbeh.2007.03.027 17493644 PMC2085359

[B27] Ravichandran KS Beginnings of a good apoptotic meal: the find-me and eat-me signalling pathways Immunity 2011 35 445 455 10.1016/j.immuni.2011.09.004 22035837 PMC3241945

[B28] Murphy JE Tacon D Tedburry PR Hadden JM Knowling S Sawamura T Peckham M Philips SE Walker JH Ponnambalam S LOX-1 scavenger receptor mediates calcium-dependent recognition of phosphatidylserine and apoptotic cells Biochem J 2006 393 107 115 10.1042/BJ20051166 16146427 PMC1383669

[B29] Strijkers GJ Hak S Kok MB Springer CS Nicolay K Three-compartment T_1_ relaxation model for intracellular paramagnetic contrast agents Magn Reson Med 2009 61 1049 1058 10.1002/mrm.21919 19215042

[B30] Kok MB Hak S Mulder WJM van der Schaft DWJ Strijkers GJ Nicolay K Cellular compartmentalization of internalized paramagnetic liposomes strongly influences both T_1_ and T_2_ Relaxivity Magn Reson Med 2009 61 1022 1032 10.1002/mrm.21910 19235908

[B31] Christiansen JP Leong-Poi H Klibanov AL Kaul S Lindner JR Noninvasive imaging of myocardial reperfusion injury using leukocyte-targeted contrast echocardiography Circulation 2002 105 1764 1767 10.1161/01.CIR.0000015466.89771.E2 11956115

[B32] Verma DD Hartner WC Levchenko TS Bernstein EA Torchilin VP ATP-loaded liposomes effectively protect the myocardium in rabbits with an acute experimental myocardial infarction Pharm Res 2005 22 2115 2120 10.1007/s11095-005-8354-x 16258743

[B33] Verma DD Hartner WC Thakkar V Levchenko TS Torchilin VP Protective effect of coenzyme Q10-loaded liposomes on the myocardium in rabbits with an acute experimental myocardial infarction Pharm Res 2007 24 2131 2137 10.1007/s11095-007-9334-0 17657597

[B34] Takahama H Minamino T Asanuma H Fujita M Asai T Wakeno M Sasaki H Kikuchi H Hashimoto K Oku N Prolonged targeting of ischemic/reperfused myocardium by liposomal adenosine augments cardioprotection in rats J Am Coll Cardiol 2009 53 709 717 10.1016/j.jacc.2008.11.014 19232905

[B35] Huynh MN Fadok VA Henson PM Phosphatidylserine-dependent ingestion of apoptotic cells promotes TGF-β1 secretion and the resolution of inflammation J Clin Invest 2002 109 41 50 11781349 10.1172/JCI11638 PMC150814

[B36] Hoffmann PR Kench JA Vondracek A Kruk E Daleke DL Jordan M Marrack P Henson PM Fadok VA Interaction between phosphatidylserine and the phosphatidylserine receptor inhibits immune responses *in vivo* J Immunol 2005 174 1393 1404 15661897 10.4049/jimmunol.174.3.1393

[B37] Ramos GC Fernandes D Charão CT Souza DG Teixeira MM Assreuy J Apoptotic mimicry: phosphatidylserine liposomes reduce inflammation through activation of peroxisome proliferator-activated receptors (PPARs) *in vivo* Br J Pharmacol 2007 151 844 850 10.1038/sj.bjp.0707302 17533418 PMC2014119

[B38] Rouser G Fleischer S Yamamoto A Two dimensional thin layer chromatographic separation of polar lipids and determination of phospholipids by phosphorus analysis of spots Lipids 1970 5 494 496 10.1007/BF02531316 5483450

[B39] Skipski VP Peterson RF Barclay M Separation of phosphatidyl ethanolamine, phosphatidyl serine, and other phospholipids by thin-layer chromatography J Lipid Res 1962 3 467 470

[B40] Garlanda C Parravicini C Sironi M De Rossi M de Calmanovici Wainstock R Carozzi F Bussolino F Colotta F Mantovani A Vecchi A Progressive growth in immunodeficient mice and host cell recruitment by mouse endothelial cells transformed by polyoma middle-sized T antigen: implications for the pathogenesis of opportunistic vascular tumors Proc Natl Acad Sci U S A 1994 91 7291 7295 10.1073/pnas.91.15.7291 8041783 PMC44385

